# Health Literacy in Context

**DOI:** 10.3390/ijerph15122657

**Published:** 2018-11-27

**Authors:** Don Nutbeam, Diane Levin-Zamir, Gill Rowlands

**Affiliations:** 1 School of Public Health, University of Sydney, Sydney 2006, Australia; 2 Department of Health Education and Promotion, Clalit Health Services, Tel Aviv 62098, Israel; diamos@zahav.net.il; 3 School of Public Health, University of Haifa, Haifa 31905, Israel; 4 Institute of Health and Society, Newcastle University, Newcastle NE1 7RU, UK; Gill.Rowlands@newcastle.ac.uk

Health literacy has been defined and conceptualized in multiple ways, but almost all definitions have similar core elements describing the personal skills that enable individuals to obtain, understand, and use information to make decisions and take actions that will have an impact on their health. These health literacy skills are not restricted in their application to personal behaviour, but can be applied to the full range of determinants of health (personal, social, and environmental). 

To date, most published health literacy research has focused on assessing and improving personal skills and abilities. More recently, a better understanding has emerged of the extent to which these skills and abilities are mediated by environmental demands and situational complexities–the context in which health literacy is developed and applied. This has led to much greater attention being given to ways of reducing the situational demands and complexity in which an individual makes a health decision. A range of models and practical strategies are emerging to help create health literate organisations. These propose strategies to reduce the environmental demands on people engaging with those organisations and health professionals. 

This Special Issue of the International Journal of Environmental Research and Public Health (IJERPH) was conceived with the aim of examining current progress in understanding health literacy in context, looking to attract papers that improve our understanding of the mutual impact of a range of social, economic, environmental, and organisational influences on health literacy.

We were especially interested in attracting submissions that reported on the relationships between physical and social environments and health literacy; interventions to reduce environmental demands and complexity, including, for example, interventions to reduce the organisational and administrative complexity of health services; health literacy interventions responsive to cultural preferences; and health literacy interventions that use the preferred media of disengaged populations.

Through the call for abstracts, we received a large number and wide range of potential submissions. This response is indicative of the high level of current interest in health literacy from a very wide range of country, sectoral, and cultural perspectives. The final group of papers selected and that completed the peer review process reflects this diversity. These papers also illustrate good progress in the evolution of research in the contexts in which health literacy is developed and applied, as well as signalling some areas in which more research would be useful.

The papers offer unique and original perspectives on the concept, distribution, and application of health literacy in very diverse populations. Several papers, including those by O’Hara et al. [1], König et al. [2], Schillinger et al. [3], Thomas et al. [4], and Lorini et al. [5], for example, offer cultural insights and a clear indication of the impact of social and environmental context on health literacy. Both individually and in combination, these findings have important implications for interventions designed to address the needs of different populations. Above all, they illustrate the need to routinely incorporate an understanding of “context” into the development of policies and programmes to improve health literacy in diverse populations. 

The papers by Trezona [6] and Geboers [7], which examine differing national policy responses to health literacy and describe a comprehensive health literacy intervention model, respectively, illustrate how policy and practice can (and should) respond to this more complete but complex understanding of health literacy. The paper by Trezona and colleagues underlines the global interest in health literacy among policy-makers, but also highlights, in turn, the gap between this public commitment and the practical actions that can be systematically applied in diverse populations.

The papers by Levin-Zamir and Bertschi [8] on the application of new digital media and the creative harnessing of popular culture (as described by Schillinger et al.) offer great promise in extending the reach and customisation of communications. These contributions, along with the papers by Rademakers et al. [9], Thomas et al. [4], and Estacio et al. [10], also demonstrate that the content of communications as well as the medium is important. 

The collection of papers published in this Special Edition includes some that focus on clinical issues (for example, McKenna et al. [11], and Stein et al. [12]), but we have also attracted a good range of papers that are community-based, give attention to the social context in which health decisions are made, and include communication content that improves our understanding of the wider social determinants of health. However, there remains a dearth of published papers that describe health literacy interventions. The great majority of published papers still focus on personal health behaviour and practices, most often in clinical settings [13].

Whilst the progress reflected in this journal is encouraging, it is evident that more discussion and research are needed to improve our understanding of health literacy in context, and how to reduce the situational demands and complexity in which an individual makes a health decision, including how organisations and social institutions can contribute. 

It is our hope that this Special Issue will be a catalyst for further action and research on health literacy in context.
